# Prevalence of birth defects and risk-factor analysis from a population-based survey in Inner Mongolia, China

**DOI:** 10.1186/1471-2431-12-125

**Published:** 2012-08-18

**Authors:** Xingguang Zhang, Su Li, Siqintuya Wu, Xiaojin Hao, Shuyi Guo, Kota Suzuki, Hiroshi Yokomichi, Zentaro Yamagata

**Affiliations:** 1 Inner Mongolia Medical College, Hohhot, China; 2 Inner Mongolia Family Planning Committee, Hohhot, China; 3 Hohhot Center of Disease Prevention and Control, Hohhot, China; 4 University of Yamanashi, Chuo-city, 409-3898, Japan

**Keywords:** Birth defects, Prevalence rate, Relative risk, Risk factors

## Abstract

**Background:**

Birth Defects are a series of diseases that seriously affect children's health. Birth defects are generally caused by several interrelated factors. The aims of the article is to estimate the prevalence rate and types of birth defects in Inner Mongolia, China, to compare socio-demographic characteristics among the children with birth defects and to analyze the association between risk factors and birth defects.

**Methods:**

Data used in this study were obtained through baseline survey of Inner Mongolia Birth Defects Program, a population-based survey conducted from 2005 to 2008. The survey used cluster sampling method in all 12 administrative districts of Inner Mongolia. Sampling size is calculated according to local population size at a certain percentage. All live births, stillbirths and abortions born from October 2005 to September 2008, whose families lived in Inner Mongolia at least one year, were included. The cases of birth defects were diagnosed by the clinical doctors according to their experiences with further laboratory tests if needed. The inclusion criteria of the cases that had already dead were decided according to death records available at local cites. We calculated prevalence rate and 95% confidence intervals of different groups. Outcome variable was the occurrence of birth defects and associations between risk factors and birth defects were analyzed by using Poisson regression analysis.

**Results:**

976 children with birth defects were diagnosed. The prevalence rate of birth defects was 156.1 per 10000 births (95%CI: 146.3-165.8). The prevalence rate of neural tube defect (20.1 per 10000 births) including anencephaly(6.9 per 10000), spina bifida (10.6 per 10000), and encephalocele (2.7 per 10000) was the highest, followed by congenital heart disease (17.1 per 10000). The relative risk (RR) for maternal age less than 25 was 2.22 (95%CI: 2.05, 2.41). The RR of the ethnic Mongols was lower than Han Chinese (RR: 0.84; 95%CI: 0.80-0.89). The RR of the third and second pregnancy was significantly higher than the first pregnancy while a slight difference between the second and the first pregnancy was also found. Alcohol drinking of mothers, familial inheritance and living area were also found to be related to the occurrence of the birth defects.

**Conclusions:**

Relatively higher birth defect rates were found in Inner Mongolia. This study found that maternal age less than 25, alcohol drinking, familiar inheritance, lower education level of mothers, times of pregnancies and living in rural areas may increase the risk of birth defects. Ethnic Mongols were less likely to have birth defects than Han Chinese.

## Background

Birth defects are defined as a series of structural, functional and metabolic disorders. According to the literatures, birth defects are a major source of infant and child morbidity and mortality and single or multiple defects can be occurred in one or several organs of the children [1]. In Western Australia, birth defects occur in 4.5% to 5.7% of live births from 2005 to 2008 [2]. Approximately 3 percent of 134 million annual births around the world are affected by a major structural disorder, a congenital abnormality [3] while the percentage of that varies among countries and regions [2-7]. In addition to the different risk factors and direct causes of birth defects seen in various parts of the world, prevalence rate also depends on the age of children, the types of malformations, the method of data collection and statistical analysis used in different studies [4-6,8]. WHO estimates prevalence of congenital disorders at 53 ‰, ranging from 40 ‰ to 50 ‰ in developed countries [7]. Birth defects are generally caused by several interrelated factors. More than half of birth defects can’t be attributed to a single factor [9]. Risk factors which contribute singly or interactively to birth defects include genetics, chemicals, physical and biological issues and maternal elements [9].

The objectives of this study are to estimate the prevalence and types of birth defects in Inner Mongolia, to compare socio-demographic characteristics among the children with birth defects and to analyze the association between risk factors and birth defects.

## Methods

Data used in this study were obtained through baseline survey of Inner Mongolia Birth Defects Program, a population-based survey conducted from 2005 to 2008. 62544 births (male: 32671; female: 29745; unknown or missing: 128) from a general population of about 600,000 over a 3 year period were selected using cluster sampling method in all 12 administrative districts of Inner Mongolia. Sampling size is calculated according to local population size at a certain percentage. All live births, stillbirths and abortions were included in screening through house to house visits by clinical doctors. Medical records including prenatal diagnostic test results, labor records, clinic diagnostic results, pathology findings, physical examinations and artificial abortions of the patients who used to seek medications at local hospitals were used as references as part of the confirmations for final diagnosis.

Two different questionnaires were developed. The first questionnaire included basic information of all eligible children such as socio-demographic factors of mothers, maternal unhealthy behaviors, history of familial inheritance, parental consanguinity, information at delivery and presence of birth defects at the time of visits. Families having a child with birth defects were asked to complete second questionnaire which contained questions on past diagnostic information at local hospitals, time duration until first diagnosis, past treatments and the types of defects.

We classified birth defects according to International Classification of Diseases 9 (ICD9). Birth defects included anencephaly (exclude acephalia and hydranencephaly), spina bifida (exclude spina bifida occulta and sacrococcygeal teratoma), encephalocele, congenital heart disease, total cleft lip (exclude median lip cleft), polydactylia (exclude bifurcate finger tip due to a broken nail), congenital hydrocephaly (exclude posteriority hydrocephaly and megacephaly without ventricle system expansion), external ear malformation, limb reduction defect, inguinal hernia, congenital hemangiomas and others. Neural tube defects included anencephaly, spina bifida, and encephalocele. Damage before and during birth was excluded from birth defects.

For data entry, we used Epidata version 3.02 (Epidata Association, Denmark) and for the statistical analysis, we used SPSS version 14.0 (SPSS Inc., Chicago,IL, USA). Median and interquartile range was calculated for continuous variables. Prevalence rate and its 95% confidence intervals (95% CI) and chi-square test were used for showing the difference on different ethnicity groups, different living areas and other predictive variables. Occurrence of birth defects is the outcome variable. We used multivariate Poisson regression analysis to show the associations between possible risk factors and birth defects. Relative risk and its 95% confidence intervals were used for multivariate analysis. P-value less than or equal to 0.05 was considered as significance using 2-tailed test.

Ethical clearance for the study was obtained from the Ethics and Research Committee of Inner Mongolia Medical College. A written informed consent was further obtained from the caregivers.

## Results

Minimum and maximum age were 16 and 48 (median: 25; interquartile range: 6) respectively. Out of 62443 births, there were 61992 live births and 451 stillbirths or abortions. Out of 976 birth defects we diagnosed in total, 164 cases were diagnosed before delivery, 575 cases were within one week after delivery, 139 cases were within one year, 44 cases were between one and two years, 29 cases were between two and three years, 5 cases were after 3 years and for 20 cases, time of the diagnosis could not be determined.

Out of 976 children diagnosed as birth defects, 294 had died due to various reasons including birth defects (crude death rate: 30.12%), while number of death was 157 (crude death rate: 0.26%) among 61568 children without birth defects. Gender distribution of the birth defects was 54.9% (535 cases) in male, 38.7% in female (378 cases), 0.6% in ambiguous (6 cases), and gender of 57 cases (5.8%) could not be recognized.

### Birth prevalence

The prevalence rate of the birth defects was 156.1 per 10000 births (95% CI: 146.3-165.8); 124.6 per 10000 births (95% CI: 111.1-138.1) in urban areas and 179.4 per 10000 births (95% CI: 165.5-193.3) in rural areas (P < 0.05).

Table 1 shows the highest prevalence rate occurred in the third or later pregnancies (513.0 per 10000 births), followed by the second pregnancy (194.1 per 10000). The first pregnancy had the lowest prevalence rate with 124.3 per 10000 (*χ*_trend_^2^ = 147.650,P = 0.000). Maternal age was divided into four different age groups: <25 years; 25–29 years; 30–35 years, and >35 years. The highest prevalence rate occurred in >35 years group followed by <25 years group. There was no statistical difference between >35 years group and <25 years group. No statistically significant difference was found among different ethnic groups (*χ*^2^=0.716,P = 0.870).

**Table 1:** Prevalence rate of different factors or characters

**Factors**	**Delivery**	**Cases**	**Prevalence rate(1/10000)**
**Ethnicity**			
Han Chinese	43503	678	155.9(144.1-167.6)
Mongolian	17051	252	147.8(129.6-166.0)
Other	1975	31	157.0(106.9-222.0)
**Living areas**			
Urban^a^	26649	332	124.6(111.1-138.1)
Rural	35895	644	179.4(165.5-193.3)
**Time of pregnancy**			
1	46186	574	124.3(114.6-134.8)
2	14426	280	194.1(172.9-218.0)
≧3	1774	91	513.0(420.1-626.1)
**Maternal age(years)**			
<25	27761	444	159.9(145.8-175.4)
25--	21098	306	145.0(129.8-162.0)
30--	9658	128	132.5(111.6-157.4)
35--	4009	80	199.6(160.8-247.8)

Table 2 shows prevalence rates of ten most common birth defects found in this study. Neural tube defect includes anencephaly (6.9 per 10000), spina bifida (10.6 per 10000) and encephalocele (2.7 per 10000). Microtia was not included in external ear malformation. Down syndrome (1.4 per 10000 births) is not listed in Table 2 because of low prevalence.

**Table 2:** Top 10 of prevalence rate of birth defects and order

**Order**	**Birth defects**	**Cases**	**Prevalence(1/10000)**
1	Neural tube defects	126	20.1
2	Congenital heart disease	107	17.1
3	Cleft lip and palate	101	16.2
4	Congenital hydrocephalus	62	9.9
5	Polydactylia	53	8.5
6	External ear malformation	27	4.3
7	Limb reduction defect	26	4.2
8	Inguinal hernia	25	4.0
9	Congenital hemangiomas	18	2.9
10	Talipes equinovarus	17	2.7

Table 3 shows the proportion of birth defects according to the location of damage in organs. The highest proportion of birth defects was seen in the central nervous system (19.8%), followed by face or eye defects (18.0%), and cardiovascular defects was 12.4%.

**Table 3:** Proportion of birth defects according to the involved organs

**Organs or systems**	**Cases**	**Proportion(%)**
Central nervous system	193	19.8
Face or eye	176	18.0
Extremities	129	13.2
Cardiovascular	118	12.1
Skeletal	76	7.8
Genitourinary tract	60	6.1
Thoracic or abdominal wall	55	5.7
Gastrointestinal tract	41	4.2
Tumors	10	1.0
Other	118	12.1
Total	976	100.0

### Risk factors analysis

We defined variables as below: ethnicity (Han Chinese, Mongols, others), maternal age (age of mother at delivery), smoking (mothers smoked more than one cigarette per day during pregnancy), alcohol drinking (alcohol volume more than 20 g at least once during pregnancy or more than 20 g per day during pregnancy), parental consanguinity (having same ancestor within 3 generations), familial inheritance history (birth defects found in family members).

### Maternal socio-demographic characteristics

The risk of birth defects was lower among ethnic Mongols than Han Chinese (RR: 0.84; 95% CI: 0.80- 0.89). The risk was higher in rural areas than urban areas (RR: 1.78; 95% CI: 1.68-1.88). There was a difference found between the maternal age groups < 25 years and >35 years (RR: 2.22; 95% CI: 2.05-2.41). Compared to mothers with highest education level, the RR of birth defects among mothers with education level less than high school was 1.69 (95%CI: 1.58-1.82) while the RR among the high school graduates was 1.06 (95%CI: 0.95-1.18). Living areas were also significantly associated with birth defects in Table 4.

**Table 4:** Association between risk factors and birth defects

**Risk factors**	**b**	**Chi-squre**	**P-value**	**RR**	**RR95%CI**
**Ethnicity**					
Han^a^	—	—	—	1.000	—
Mongolian	−0.172	41.05	0.000	0.842	0.799-0.888
Other	0.008	0.01	0.905	1.008	0.888-1.138
**Maternal age (years)**					
<25	0.798	374.28	0.000	2.220	2.048-2.408
25--	0.514	176.03	0.000	1.672	1.550-1.805
30--	0.227	20.34	0.000	1.255	1.137-1.385
35--^a^	—	—	—	1.000	—
**Smoking**					
No^a^	—	—	—	1.000	—
Yes	−0.129	2.12	0.145	0.879	0.736-1.041
**Drinking**					
No^a^	—	—	—	1.000	—
Yes	1.169	55.65	0.000	3.220	2.332-4.319
**Time of pregnancy**					
1	−1.769	1603.35	0.000	0.171	0.156-0.186
2	−1.017	579.74	0.000	0.362	0.333-0.393
≧3^a^	—	—	—	1.000	—
**Parental consanguinity**					
No^a^	—	—	—	1.000	—
Yes	0.305	3.73	0.053	1.356	0.981-1.822
**Familial inheritance history**					
no^a^	—	—	—	1.000	—
yes	2.413	1480.85	0.000	11.165	9.852-12.600
**Maternal education**					
Less than high school	0.526	214.23	0.000	1.692	1.577-1.815
High school	0.059	1.17	0.279	1.061	0.952-1.180
More than high school^a^	—	—	—	1.000	—
**Living area**					
Urban^a^	—	—	—	1.000	—
Rural	0.576	393.87	0.000	1.779	1.681-1.884

^a^: reference.

### Maternal birth history and unhealthy behaviors during pregnancy

Drinking alcohol was associated significantly with the risk of birth defects while no association was found between smoking and birth defects. Families with familial history of birth defects were more likely to have birth defects (RR: 11.17; 95% CI: 9.85, 12.60) than those without family history. Times of pregnancy was also associated with the occurrence of birth defects in Table 4.

## Discussion and conclusions

Compared to previous studies in China, this study had larger sample size to estimate prevalence rate and to explore the associations between possible risk factors and birth defects by controlling for other predictive variables respectively through multivariate analysis. The prevalence rate of birth defects in Inner Mongolia was 156.1 per 10000 births (95%CI: 146.3-165.8 per 10000), which is consistent with the prevalence rate in Gansu Province, western China (154.0 per 10000 births) [10]. The prevalence rate of neural tube defect was found to be the highest (20.1 per 10000) in this study, which was shown to be higher than that in other provinces of China (15.9 per 10000) and even higher than the prevalence in Korea (5.1 per 10000) [11,12]. The prevalence of cleft lip and palate together and congenital hydrocephalus in Inner Mongolia (16.2 per 10000 and 9.9 per 10000, respectively) were also higher than the prevalence in Korea (10.3 per 10000 and 3.6 per 10000, respectively) and some provinces of China (13.6 per 10000 and 6.5 per 10000, respectively) [11,12]. The prevalence of congenital heart disease in this study (17.1 per 10000) was lower than in Belgium (83.0 per 10000) [13], but similar to the prevalence in Colombia (12.0 per 10000) [14]. One reason for the lower prevalence of congenital heart disease may due to the diagnostic criteria which only included severe congenital heart disease in this study, which is consistent with the criteria used in a study in Taiwan (14.2 per 10000) [15]. The difference of prevalence rates among different regions either in China or other countries may due to the period children were observed after birth, the types of birth defects, different data collection method, method of statistical analysis [4-6,8].

There might be several possible reasons for the higher risk of birth defects we observed in families living in rural areas. First, living, and especially working, in rural areas may bring people into contact with substances related to birth defects that are not present in urban areas. The results of one study indicated an association between birth defects and maternal agricultural work [16,17]. Second, different lifestyles and habits of mothers or their living conditions or taking nutrition supplements may differ between rural and urban areas, for example, whether the use of multivitamin supplements is promoted during pregnancy or not in rural areas. A study showed that multivitamin and folic acid supplementation before the pregnancy can reduce the overall occurrence of congenital abnormalities and neural-tube defects [3]. The RR of birth defects among mothers with prenatal alcohol exposure was 3.22 (95% CI: 2.33-4.32) in this study. The result is slightly differed from a study which reported an adjusted odds ratio of 4.6 (95% CI: 1.5-14.3) for mothers with heavy prenatal alcohol exposure in the first trimester of pregnancy [18].

In this study, we found no association between prenatal smoking habits of mothers and birth defects. The result is similar to a study showed that the offspring exposed to prenatal tobacco smoke had no increase in the prevalence of congenital malformations compared with non-exposed offspring in both crude and adjusted analyses [19].

This study found that younger mothers aged <25 years had a greatly increased risk of birth defects in their offspring, which is consistent with a research in China [20]. The authors found a statistically significant difference in prevalence of birth defect among different maternal education levels. This difference may indirectly duo to mothers’ socioeconomic status, dietary habits, neighborhood conditions, and access to health care and appropriate foods, as well as the mother’s knowledge about the importance of folic acid in the diet [21-23]. Previous studies showed that offspring with parental consanguinity had an increased risk for major birth defects [24,25], but parental consanguinity was not predictive in this study. Given a history of similar defects in first-degree relatives, the relative risk of birth defects increased from a study of Denmark [26]. Besides, another study of Xinjiang Autonomous Region showed that the risk of birth defects was higher among ethnic Han Chinese than other ethnicities [27]. The results in this study also indicated that familial inheritance and ethnicity were related to birth defects.

The following is the limitations of the study: 1) it may could not include all cases with birth defect due to the limitations of diagnostic methods, for example, defects with insignificant symptoms are not likely to be found in early years of childhood; 2) perinatal death prior to diagnosis was also not included in this study.

Through this study, it found lower risk of birth defects in Mongolian than Han Chinese and other ethnic groups, which need further research on the influence of genetics or cultural and environmental factors on birth defects among Mongols. This study also found that maternal age less than 25, alcohol drinking, familiar inheritance, lower education level of mothers, times of pregnancies and living in rural areas may increase the risk of birth defects. Moreover, some environmental factors such as underground water contamination due to mining activities, agriculture chemical exposure such as overdose using of pesticides etc. should be explored in the future. Based on the results which was obtained in this study, a few intervention activities are recommend as follows: 1) regular screening tests among pregnant women are needed at local health care sectors to decrease the rate of birth defects in Inner Mongolia; 2) in rural areas, health education programs among females at reproductive age and among pregnant women are also necessary to avoid exposures to preventable risk factors of birth defects; 3) health education for pregnant women at younger age both in rural and urban areas should be taken into consideration as part of the interventions at their first visit to the hospitals.

## Competing interests

The authors declare that there are no personal, organizational or financial conflicts of interest.

## Authors’ contributions

XZ, SL, SW and XH participated in the planning, conception of the research questions, and design of the study. XZ, SL, XH and SG organized the investigation of the study and analyzed the data. XZ, KS, HY and ZY drafted the article and participated in interpreting data and critically revising the manuscript for important intellectual content. All authors read and approved the revised manuscript.

## Pre-publication history

The pre-publication history for this paper can be accessed here:

http://www.biomedcentral.com/1471-2431/12/125/prepub
